# The Role of Ancient Greek Physicians in the Development of Tracheostomy: Pioneering Airway Interventions and Early Thoracic Surgery

**DOI:** 10.3390/clinpract15050093

**Published:** 2025-05-13

**Authors:** Vasileios Leivaditis, Francesk Mulita, Nikolaos G. Baikoussis, Elias Liolis, Andreas Antzoulas, Levan Tchabashvili, Konstantinos Tasios, Dimitrios Litsas, Manfred Dahm

**Affiliations:** 1 Department of Cardiothoracic and Vascular Surgery, Westpfalz Klinikum, 67655 Kaiserslautern, Germany; vnleivaditis@gmail.com (V.L.); mdahm@westpfalz-klinikum.de (M.D.); 2 Department of General Surgery, General Hospital of Eastern Achaia—Unit of Aigio, 25100 Aigio, Greece; tchabashvili.alexander@gmail.com; 3 Department of Cardiac Surgery, Ippoktration Gernaral Hospital of Athens, 11527 Athens, Greece; nikolaos.baikoussis@gmail.com; 4 Department of Oncology, University Hospital of Patras, 26504 Patras, Greece; lioliselias@yahoo.gr; 5 Department of General Surgery, University Hospital of Patras, 26504 Patras, Greece; a.antzoulas@hotmail.com (A.A.); kostastasiosmd@gmail.com (K.T.); 6 Department of General Surgery, General Hospital of Lamia, 35131 Lamia, Greece; dimlitsas@icloud.com

**Keywords:** tracheostomy, ancient Greek medicine, airway management, thoracic surgery, Hippocratic medicine, Galen of Pergamon, surgical history

## Abstract

Tracheostomy, a critical airway intervention, has a long and complex history that dates back to antiquity. While the earliest references to the procedure appear in Egyptian and Indian medical texts, its development within ancient Greek medicine remains a subject of historical debate. This study explores the evolution of tracheostomy in ancient Greece, analyzing its theoretical foundations, historical accounts, and surgical advancements. Despite Hippocratic opposition, which largely discouraged invasive airway procedures due to the risk of fatal complications, later physicians such as Asclepiades, Aretaeus, and Antyllus made significant contributions to refining airway management techniques. The anatomical studies of Galen further advanced the understanding of respiratory physiology, including early concepts of artificial ventilation. Additionally, this study examines archaeological evidence, such as a marble relief discovered in Abdera, which may depict an early attempt at tracheostomy, providing valuable insight into the practical application of airway interventions in antiquity. By comparing ancient Greek surgical techniques with modern tracheostomy practices, this research highlights the continuity of medical knowledge and innovation. It underlines the role of ancient Greek physicians in shaping the principles of thoracic surgery, offering a broader understanding of how early medical practices have influenced contemporary airway management. The findings contribute to the historical perspective on tracheostomy, emphasizing the timeless pursuit of life-saving surgical advancements and the evolving relationship between theoretical medical knowledge and practical surgical application.

## 1. Introduction

The history of surgical procedures can be traced back to antiquity, with ancient Greece serving as one of the earliest centers of medical knowledge and innovation. The medical texts of Greek, Roman, and Arabic antiquity offer valuable insights into the evolution of surgical techniques, the cultural and philosophical foundations of healing, and the growing body of anatomical and physiological knowledge that shaped early medical practice. Physicians such as Hippocrates (c. 460–370 BCE), Herophilus of Chalcedon (c. 335–280 BCE), and Galen of Pergamon (129–199 CE) laid the groundwork for surgical interventions, including procedures related to the lungs, airway management, and thoracic structures [1,2,3].

Among the most critical thoracic interventions was tracheotomy, a procedure designed to secure the airway in cases of life-threatening obstruction. Although the technique carried significant risks, its life-saving potential made it an enduring subject of medical discourse in antiquity. The practice of early thoracic surgery, particularly its role in ensuring adequate oxygen supply and respiratory function, is reflected in various ancient medical writings and historical accounts. Beyond tracheotomy, ancient physicians explored rudimentary forms of ventilation and paracentesis to relieve conditions such as mediastinal effusions, pneumothorax, or respiratory distress, foreshadowing modern thoracic surgical interventions [4,5].

Despite opposition from prominent figures such as Hippocrates, who deemed tracheotomy excessively dangerous due to the risk of injuring major blood vessels, later physicians—particularly those in Hellenistic and Roman medicine—experimented with refinements of the technique. Figures such as Asclepiades of Bithynia, Antyllus, and Galen contributed to the understanding of airway anatomy, surgical techniques, and postoperative care, some of which influenced later medieval and Renaissance surgical practices. Additionally, historical texts suggest that emergency airway interventions were performed not only in medical settings but also on the battlefield, where swift action was required to save lives [6,7,8,9].

The study of early thoracic surgery and tracheotomy provides valuable perspectives on the progression of surgical techniques and their transition into modern thoracic and airway management practices. From an evidence-based standpoint, analyzing historical surgical interventions enhances our appreciation of decision-making processes in medicine, demonstrating how ancient physicians navigated anatomical challenges with limited instrumentation and knowledge. Even in contemporary thoracic surgery, historical cases serve as teaching tools, illustrating the evolution of surgical innovation, anatomical discovery, and patient care over centuries [10,11,12].

This article examines the historical development of tracheotomy and other early thoracic surgical interventions, analyzing their medical rationale, technical execution, and legacy in modern surgical practice. By exploring ancient Greek, Roman, and early Islamic medical traditions, we can better understand how early surgical pioneers contributed to the foundations of modern airway management and thoracic surgery.

### 1.1. Background and Significance

Tracheostomy is one of the oldest surgical procedures performed on the thoracic cavity, with its earliest documented reference found in the *Sushruta Samhita*, an ancient Indian text regarded as the earliest known treatise on surgery and medicine [3,9,13,14]. This foundational work from India marks the beginning of documented surgical procedures, and tracheostomy is one of the earliest recorded interventions within its scope. However, the Greek literature offers significant insights into the historical development of tracheostomy within the Western medical tradition, particularly through references dating back to Hippocrates and his contemporaries.

While the concept of tracheostomy was not widely documented in the earliest Greek texts, its presence in medical writings from the fifth century BCE provides an essential understanding of how early Greek physicians approached airway management. Notably, references to this procedure are explored within the broader context of Greek surgical practices which, until the Hellenistic period, were often considered secondary to other healing practices. The status of surgery in ancient Greece was subordinated to fields like philosophy, ritual healing, and herbal medicine, despite continuous advancements in surgical techniques [9,15,16,17].

The role of Greek surgical interventions improved significantly during the Hellenistic era. It was during this period that Hippocrates and Herophilus made pivotal contributions, laying the groundwork for systematic anatomical studies and surgical practices [18]. While Herophilus is renowned for his pioneering anatomical studies, historical accounts suggest that his dissections and vivisections were primarily conducted on animals and, according to some sources, on condemned criminals, as broader human dissection was socially and legally restricted at the time. Yet, it was under Roman dominance that the status of surgery and the role of surgeons saw a major transformation. Roman medicine, influenced by Greek knowledge, began to formalize surgical practices, recognizing the importance of surgical procedures like tracheostomy in the treatment of airway obstructions. The contributions of physicians such as Galen further solidified surgery’s standing as a respected medical discipline [19].

The historical significance of tracheostomy as one of the earliest thoracic interventions is highlighted not only by these medical advancements but also through its documentation in ancient medical texts. Despite its origins in ancient Indian and Greek traditions, it is clear that the Greek literature of the fifth century BCE represents the first written mentions of tracheostomy in the Western world. This marks an important moment in the history of surgical intervention for respiratory conditions [9,14,17].

Although *The Iliad*, Homer’s epic detailing the wounds of warriors, has been extensively analyzed by modern medical scholars, tracheostomy is notably absent from the surgical interventions described within [20]. While other poets, such as Hesiod and the authors of the *Argonautica*, mention medical and surgical practices, there are no references to tracheostomy in their works. This suggests that, despite the presence of other surgical interventions in these texts, tracheostomy was not yet widely practiced or understood in the ancient Greek world [21].

Moreover, despite the primitive medical technology and limited knowledge of anatomy in ancient times, tracheostomy was rarely applied in practice, even in situations where airway management was critical. Historical accounts do mention various surgical techniques used to treat obstructions, such as the use of inflatable bladders and the insertion of sponge or ceramic tubes into the trachea to facilitate breathing [22]. However, these procedures were not consistent or widely adopted, and tracheostomy as a standardized practice was not fully realized in the Greek or Roman medical traditions. As such, these early efforts can be seen as precursors to the more formalized practices of airway management that would emerge in later centuries, particularly in the context of modern thoracic surgery [5,23,24,25,26].

The ingenuity of ancient physicians, their methods of airway management, and their contributions to surgical practice cannot be understated. Although tracheostomy was not widely recognized or standardized in the ancient world, these early efforts provided the foundation for future developments in respiratory medicine and surgical techniques. Their contributions are a testament to the evolution of surgical knowledge and the continued development of medical practices across the centuries [9,16,17].

### 1.2. Purpose and Scope of the Study

The study of tracheostomy and early thoracic surgery in ancient Greece remains a largely underexplored topic, with only limited scholarly engagement in primary sources and historical medical literature [27,28,29]. This research aims to investigate whether ancient Greek physicians developed or performed early forms of tracheostomy or other thoracic surgical procedures. By analyzing primary medical texts, historical references, and linguistic evidence, this study seeks to determine the importance, relevance, and potential modern applications of these ancient practices.

A key objective of this research is to establish connections between ancient and modern surgical knowledge, tracing the lines of transmission from Greek antiquity to contemporary thoracic and airway management techniques. This requires a multifaceted approach, involving:

- The examination of medical procedures recorded in Greek, Roman, and later medical traditions.
- The analysis of surgical terminology, including etymological insights into medical terms related to airway intervention and thoracic surgery.
- The assessment of historical diagnostic methods, surgical techniques, and their evolution over time.
- The investigation of ancient medical instruments used for airway interventions and their technological significance.
- The evaluation of ancient descriptions of conditions that might have warranted surgical intervention, with a focus on their relevance to modern pathology and surgical principles.

Additionally, this study explores historical instances of emergency airway management, particularly in cases where tracheostomy or intubation may have been attempted as a last-resort effort. This includes an analysis of whether such procedures were performed in cases of strangulation or respiratory distress, as well as their role in battlefield medicine and civilian healthcare.

Furthermore, this study critically examines existing scholarly perspectives on early Greek surgical practices and integrates newly uncovered historical evidence that may contribute to a more comprehensive understanding of ancient thoracic surgery. Even though recent research does not always directly address tracheostomy, certain works provide valuable contextual information that enhances this study’s broader scope [30,31].

By systematically reviewing primary texts, linguistic records, and archaeological findings, this research aims to bridge the gap between ancient and modern surgical knowledge, offering new insights into the historical development of airway management and thoracic interventions.

## 2. Materials and Methods

### 2.1. Study Design and Approach

This study employs a historical-analytical approach to examine the origins, development, and implications of tracheostomy in ancient Greek medicine. By analyzing primary historical texts, archaeological findings, and secondary academic literature, this research aims to reconstruct the knowledge and surgical practices related to early airway management and thoracic surgery. This study compares ancient Greek medical techniques with those of other civilizations and explores their relevance to modern surgical practices.

### 2.2. Sources and Data Collection

Secondary sources were identified through searches in major academic databases, including PubMed, Google Scholar, and Scopus, using terms such as ‘tracheostomy history’, ‘ancient Greek surgery’, and ‘early airway management’. Recent peer-reviewed books and historical commentaries were also consulted to provide a comprehensive and updated context for interpreting ancient medical practices. This research draws upon a multidisciplinary collection of sources, including the following:

Primary ancient texts

- The Hippocratic Corpus (5th–4th century BCE);
- Writings of Galen of Pergamon (2nd century CE);
- Medical treatises by Aretaeus of Cappadocia, Antyllus, and Asclepiades of Bithynia;
- Other ancient Greek references to airway surgery and thoracic interventions;

Archaeological evidence

- The Abdera marble relief, which is hypothesized to depict an early form of tracheostomy;
- Ancient surgical instruments associated with airway procedures and thoracic interventions;
- Comparative artifacts from Egyptian, Indian, and Roman medical practices;

Secondary literature and modern analysis

- Peer-reviewed journal articles on the history of tracheostomy and thoracic surgery;
- Books and historical analyses of Greek medicine and surgical advancements;
- Modern medical perspectives on the evolution of airway management techniques.

### 2.3. Methodology

Textual analysis

- A systematic review of primary medical texts was conducted to identify references to tracheostomy, airway obstruction management, and surgical interventions.
- Translations of ancient Greek medical writings were examined for descriptions of tracheal procedures, surgical tools, and treatment of respiratory conditions.

Comparative historical analysis

- This study compares Greek tracheostomy techniques with those found in Egyptian and Indian traditions, as well as later Roman and Islamic medical advancements.
- The anatomical and surgical knowledge of ancient Greek physicians was assessed in relation to contemporary thoracic surgery principles.

Archaeological and artifactual interpretation

- The Abdera marble relief was analyzed within the context of ancient Greek medical practices and compared with other depictions of surgical interventions.
- References to surgical instruments and their potential use in early airway procedures were evaluated based on historical records and modern reconstructions.

### 2.4. Limitations and Considerations

This study is limited by the availability and interpretability of ancient sources, as some primary texts have been lost or survive only in fragmentary form. Additionally, while the Abdera relief offers intriguing evidence, its exact interpretation as a tracheostomy depiction remains hypothetical. The lack of direct experimental validation of ancient surgical techniques also presents a challenge, as reconstructions rely on textual descriptions and artifacts rather than practical demonstrations.

### 2.5. Ethical Considerations

As this study is based on historical and literary sources, no ethical approval was required. The research adheres to academic integrity standards by ensuring accurate citation and attribution of all primary and secondary sources.

## 3. Definition and Purpose of Tracheostomy

### 3.1. Definition

Tracheostomy is a surgical procedure performed in cases where the airway is obstructed, when prolonged mechanical ventilation (beyond 7–14 days) is required, or as a prophylactic measure to ensure airway patency. The procedure involves the creation of a temporary or permanent opening in the trachea to provide direct access to the airway, facilitating mechanical ventilation and respiratory support. It is important to note the etymological distinction between the terms tracheotomy and tracheostomy, both derived from ancient Greek. The word *τομή* (-tomy) refers to ‘cutting’ or ‘incision’, thus tracheotomy specifically denotes the surgical act of making an incision into the trachea. In contrast, *στομία* (-stomy) means ‘mouth’ or ‘opening’, and tracheostomy refers to the creation of a semi-permanent or permanent opening (stoma) into the trachea, typically maintained with a tube for ongoing airway access. While the two terms are often used interchangeably in modern literature, especially in clinical settings, the distinction is important in historical and surgical discussions.

Historically, tracheostomy has evolved in response to epidemics, traumatic injuries, and wartime medical challenges. It is primarily indicated for patients who are unable to breathe through the upper airway due to obstruction, reduced airway patency, or neuromuscular impairment [32,33,34,35,36].

Upper airway obstruction can result from various conditions, including the following:

- Trauma (e.g., maxillofacial fractures, laryngeal injuries, etc.);
- Malignancies (e.g., upper airway or laryngeal cancers causing stenosis);
- Severe infections (e.g., epiglottitis, deep neck infections, etc.);
- Neuromuscular disorders (e.g., ALS, myasthenia gravis, or prolonged intubation-related failure to wean);
- Palliative care settings, where long-term ventilation is required at the end of life.

### 3.2. Purpose and Importance

Tracheostomy is a highly invasive surgical procedure that requires a high level of technical skill and anatomical understanding to perform safely. One of the most critical and life-threatening complications is sudden airway obstruction, which can lead to hypoxia, respiratory distress, and fatal consequences if not promptly addressed. Patients experiencing acute airway blockage often exhibit severe respiratory distress and panic, and failure to re-establish airway patency can result in irreversible hypoxemia, hypercapnia, and acidemia, leading to respiratory failure and death [32,37,38,39].

Maintaining airway patency is one of the most fundamental yet challenging aspects of tracheostomy management. Key concerns include the following:

- Tracheal stenosis: chronic trauma and granulation tissue formation at the stoma site can lead to airway narrowing, requiring frequent surveillance and possible surgical revision.
- Mechanical damage: high vacuum pressure during suctioning combined with excessive movement of the tracheostomy tube can cause tracheal injury.
- Secretion management: a large suction load is often required to clear mucus buildup, reducing the risk of obstruction and infection.
- Long-term complications: chronic tracheostomy use may result in tracheomalacia, tracheoesophageal fistula formation, or chronic inflammation of tracheal mucosa.

The ability to effectively maintain tracheostomy patency and prevent life-threatening complications requires continuous monitoring, specialized airway care, and a multidisciplinary approach. Advances in modern tracheostomy techniques have significantly reduced mortality and improved patient outcomes, yet the procedure remains one of the most delicate and complex surgical interventions in airway management [39,40,41].

## 4. Historical Context

### 4.1. Medical Practices in Ancient Greece

Ancient Greek medicine was a blend of mythological beliefs, empirical observations, and emerging scientific methodologies. The earliest notions of healing were deeply rooted in religious and supernatural traditions, with Hephaistos, the god of craftsmanship and metallurgy, sometimes considered an early divine practitioner of medicine. Asclepius, the Greek god of medicine, was revered as a divine healer and the son of Apollo, embodying both mythological and medical significance in ancient Greece. His daughters, Hygieia, Panacea, Aceso, Iaso, and Aegle, became associated with various aspects of healing—Aegle symbolizing beauty and well-being, Panacea representing universal remedies, and Hygieia personifying cleanliness and disease prevention [42].

**Figure 1:**
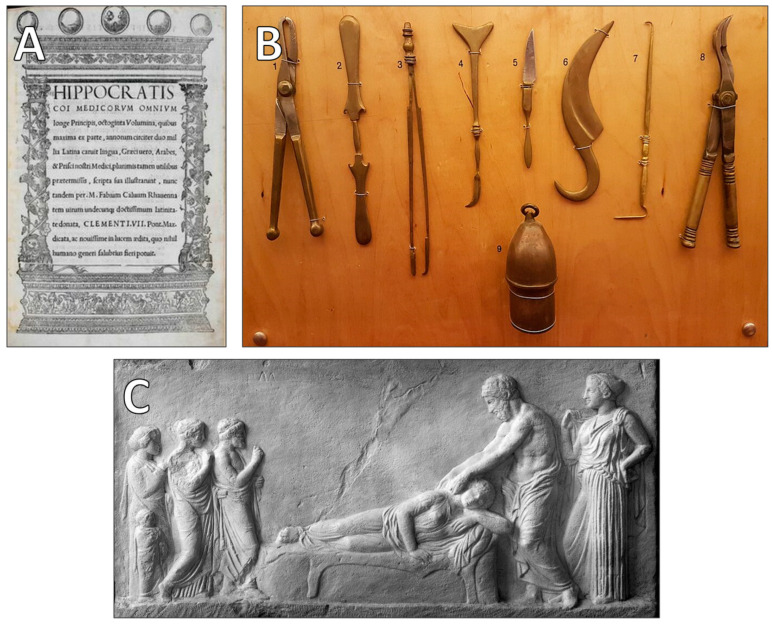
Historical snapshots illustrating the legacy and sophistication of ancient Greek medicine. (**A**) Title page from the *Octoginta volumina* (1525, Rome), representing the first comprehensive Latin translation of the *Hippocratic Corpus*. This pivotal publication facilitated the dissemination of Hippocratic medical teachings throughout Renaissance Europe, significantly shaping modern medical thought. (**B**) Modern reconstruction of ancient Greek surgical instruments dating to the 5th century BCE, derived from detailed textual descriptions in the Hippocratic Corpus and exhibited at the Thessaloniki Technology Museum. These tools exemplify the advanced practical knowledge and technical proficiency achieved by early Greek surgeons. (**C**) Classical marble relief from an Athenian temple illustrating the compassionate interaction between a physician and a female patient. This artifact highlights the integral societal role of medical practitioners in ancient Greece and underscores the enduring humanistic foundations of clinical care (Credit: Wellcome Images/Wikimedia Commons).

As medical thought progressed, temples of Asclepius (Asklepieia) emerged as centers of medical learning and healing. These sanctuaries, dedicated to Asclepius, the god of medicine, functioned as both spiritual and practical medical institutions, where priests and physicians provided treatments ranging from herbal remedies to minor surgical interventions. The Hippocratic tradition, which began in the fifth century BCE, further transformed medical practice by advocating a rational, observation-based approach to diagnosis and treatment, emphasizing the natural causes of disease rather than divine punishment [43,44,45,46,47]. The enduring legacy of these medical advancements, alongside representative examples of ancient Greek medical literature, instruments, and patient care practices, is highlighted in Figure 1.

Greek medical philosophy evolved through critical inquiry and debate. The early philosophers and physicians—including Hippocrates, Aristotle, and Herophilus—sought to explain bodily functions using systematic observations rather than supernatural interpretations. Hippocratic texts introduced the concept of disease etiology, and Greek physicians began classifying illnesses based on symptoms and progression [6]. Porphyry, a Neoplatonist philosopher, noted the philosophical and observational roots of medicine, describing how early thinkers experimented with theriacs (antidotes) and various treatments long before formalized medical knowledge existed. Over time, the male physician replaced the traditional female prophetess, shifting medical practice from a spiritual realm to a structured, evidence-based discipline. The doctor–patient relationship was built on mutual respect, a principle reinforced by the Hippocratic Oath, which sanctified medical ethics and professional duty [48,49,50].

### 4.2. Evidence of Early Thoracic Interventions

The exact origins of tracheostomy remain uncertain, and historical records provide little clarity on the first successful procedure. However, ancient medical texts indicate that Greek physicians had a sophisticated understanding of the trachea and its associated pathologies. They managed severe airway obstructions, traumatic injuries, and infections affecting the respiratory tract. Early descriptions of bronchial fistulas date back to 3300 BCE, while texts from circa 1500 BCE document treatments for tracheal and bronchial infections, as well as references to tracheal ulcers [32,51].

Despite their knowledge of airway anatomy, ancient physicians faced significant challenges in performing tracheostomy safely. Before the discovery of anesthesia (ether-based or otherwise), pain control was limited, and most surgical procedures had to be conducted rapidly to minimize patient distress and blood loss. While tracheostomy itself is a relatively swift procedure, the real challenge lay in securing the airway postoperatively, particularly in maintaining a patent tracheal opening in the absence of cannulas or modern tracheostomy tubes. Ancient physicians lacked specialized airway management tools, which made postoperative care exceedingly difficult [52,53,54].

In the absence of tracheostomy tubes, ancient practitioners faced several complications, including the following:

- Hemorrhage: vascular injury, particularly to the inferior thyroid artery and adjacent vessels, often led to fatal bleeding.
- Mucus obstruction: the accumulation of secretions in the airway frequently resulted in asphyxiation.
- Infections: without antiseptic techniques, tracheal incisions were highly prone to secondary infections.
- Lack of airway maintenance: the inability to keep the tracheal opening patent led to rapid closure of the incision site, rendering the procedure ineffective.

In some cases, ancient medical practitioners attempted alternative approaches when tracheostomy failed. When tracheostomy failed or was considered too dangerous to perform, ancient physicians occasionally attempted alternative supportive measures. These included non-invasive techniques such as steam inhalation to loosen secretions, body repositioning to facilitate airflow, and herbal remedies aimed at reducing inflammation or soothing the throat. In more extreme cases, gastrostomy was performed not to address respiratory distress directly, but to prolong survival in patients unable to eat due to upper airway obstruction. If a patient was unable to breathe due to airway obstruction, some sources suggest that a gastrostomy might have been performed to prolong survival by introducing nutrition directly into the stomach. In these early cases, feeding following gastrostomy was typically carried out using hollow reeds, metal tubes, or other primitive conduits inserted into the stomach, allowing the administration of broths or herbal liquids. Although poorly standardized and high-risk by today’s standards, such attempts reflected the early physicians’ ingenuity in supporting nutrition when oral intake was impossible. While this measure did not address respiratory failure, it was one of the few available life-sustaining interventions in cases of severe upper airway obstruction. These methods demonstrate the resourcefulness of early medical practitioners working with limited anatomical and technological knowledge [55].

### 4.3. Ancient Greek Contributions to Airway Surgery

Although tracheostomy was rarely performed, Greek physicians made significant contributions to airway anatomy and thoracic surgery. Some of the most influential figures include the following:

- Hippocrates (460–370 BCE): while he opposed tracheostomy due to the high risk of fatal complications, his medical treatises contributed to the understanding of airway diseases and surgical ethics.
- Herophilus of Chalcedon (c. 335–280 BCE): a pioneering anatomist and surgeon, Herophilus performed dissections and described the larynx and trachea, advancing knowledge of respiratory physiology.
- Asclepiades of Bithynia (124–40 BCE): credited with one of the earliest recorded tracheotomies, Asclepiades proposed that a controlled incision in the trachea could alleviate airway obstruction.
- Galen of Pergamon (129–199 CE): his detailed anatomical descriptions of the trachea, lungs, and larynx laid the groundwork for future respiratory medicine. He was also the first to document experiments in artificial ventilation, using bellows to inflate the lungs of animals, demonstrating an early understanding of mechanical ventilation [32,51,52].

### 4.4. Limitations and Challenges in Ancient Airway Surgery

While Greek and Roman physicians had an impressive grasp of anatomy and pathology, their ability to effectively treat airway obstruction was severely limited by the following:

- Lack of anesthesia and antiseptic techniques, making surgery highly painful and infection-prone;
- Absence of effective surgical instruments, particularly for airway stabilization;
- Limited understanding of postoperative care, leading to high mortality rates;
- Skepticism surrounding surgical interventions, as many Greek physicians favored non-invasive treatments over direct surgical intervention [53,54].

Despite these obstacles, Greek medical traditions influenced future generations of physicians, particularly during the Islamic Golden Age (8th–13th centuries), when Greek medical texts were translated, studied, and expanded upon by scholars such as Avicenna and Al-Razi. These texts would later inspire Renaissance advancements in surgery, ultimately leading to the refinement of tracheostomy techniques in the 19th and 20th centuries [55].

## 5. Tracheostomy in Ancient Greece

### 5.1. Early Origins of Tracheostomy in Ancient Civilizations

**Figure 2:**
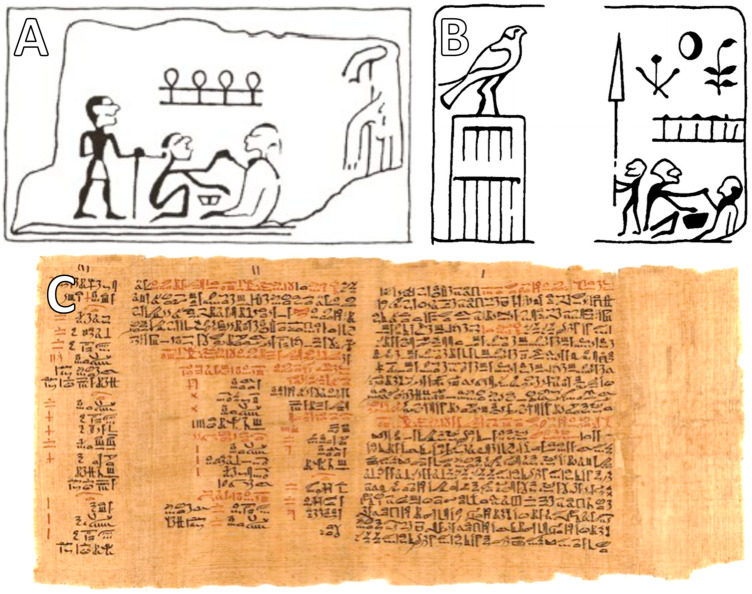
Early Egyptian representations potentially related to tracheotomy or airway interventions. (**A**) Fragment of a tablet from Abydos, dated to approximately 3600 BCE, believed by some scholars to illustrate an early medical act involving the neck or airway region. While its exact interpretation remains debated, the image is often cited as suggestive of primitive surgical awareness. (**B**) Relief from the reign of King Aha (ca. 3100 BCE), 1st Dynasty, reproduced from Pahor AL (*Ear, Nose and Throat in Ancient Egypt*, J Laryngol Otol. 1992;106:773–9) [57]. The scene has been interpreted as depicting a form of neck intervention, possibly ritualistic or therapeutic in nature (courtesy of *The Journal of Laryngology and Otology*). (**C**) Segment of the Ebers Papyrus (ca. 1550 BCE), one of the oldest known medical texts, which includes descriptions of conditions involving the throat and respiratory system. Though it does not directly describe tracheostomy, its detailed anatomical observations and therapeutic strategies underscore the Egyptians’ advanced interest in managing airway-related illnesses.

The origins of tracheotomy date back to some of the earliest recorded medical practices in human history. Egyptian medicine provides the first known depictions of this procedure, with two ancient Egyptian tablets dating from approximately 3600 BCE. Possible depictions of early airway interventions in ancient Egypt, dating as far back as 3600 BCE, are illustrated in Figure 2, including a tablet from Abydos and a ceremonial slab from the reign of King Aha, which some scholars interpret as representing primitive tracheotomy-like procedures [3,5]. Further evidence of early airway interventions can be found in the Ebers Papyrus (circa 1550 BCE), one of the most comprehensive Egyptian medical manuscripts, which references surgical techniques that suggest knowledge of tracheotomy [5,7]. Ancient Indian texts also contain significant mentions of airway procedures. The *Rigveda*, a sacred Hindu scripture, describes a figure capable of reuniting the windpipe without a ligature, provided the cervical cartilages are not completely severed [56]. Another significant Indian text, the *Sushruta Samhita* (circa 400 BCE), which is fundamental to Ayurvedic medicine and surgery, explicitly discusses tracheotomy techniques, demonstrating that the procedure was not unknown in early medical traditions across different civilizations [17].

### 5.2. Tracheostomy in Ancient Greek Literature and Historical Accounts

The term ‘tracheostomy’ originates from the Greek word τραχεία (tracheia), meaning ‘rough’, referring to the textured surface of the tracheal cartilage. Ancient Greek texts describe the trachea as a continuation of the mouth, leading to both the lungs and stomach [58,59,60]. Although direct mentions of tracheostomy remain scarce in surviving Greek medical texts, various references to airway management and surgical interventions exist [32,54,60,61]. Hippocrates, Theophilus, Celsus, Soranus, and Galen all described medical conditions that could necessitate airway intervention [32].

Although tracheostomy was not widely performed in ancient Greece, Greek physicians laid the theoretical foundations for later advancements in airway surgery. The prevailing skepticism toward the procedure, largely influenced by Hippocratic thought, delayed its widespread acceptance, but later physicians in the Hellenistic and Roman periods began refining its techniques [62,63]. The anatomical studies of Galen, the surgical contributions of Antyllus, and the experimental approaches of various Greek and Roman medical scholars collectively contributed to the gradual development of airway management techniques. These early medical insights, though rudimentary by modern standards, highlight the enduring legacy of ancient Greek medicine in shaping contemporary surgical practices [17].

## 6. Key Hellenic Figures in Tracheostomy Development

### 6.1. Hippocrates and the Opposition to Tracheostomy

Hippocrates of Kos (c. 460–370 BCE), widely regarded as the father of Western medicine, played a pivotal role in shaping ancient Greek surgical philosophy. However, his influence greatly discouraged the practice of tracheostomy, primarily due to the perceived risks associated with the procedure. His medical teachings, compiled in the Hippocratic Corpus, emphasize a cautious and conservative approach to surgery, prioritizing non-invasive treatments over aggressive interventions [3,12].

Hippocrates strongly warned against the danger of severe hemorrhage that could result from inadvertently severing the carotid artery or major blood vessels during a tracheal incision. At the time, Greek physicians lacked a precise understanding of vascular anatomy, making blood loss a leading cause of mortality in surgery. Additionally, Hippocrates noted the difficulty of treating fistulas in cartilaginous regions, explaining that such wounds often failed to heal properly, which made the operation not only dangerous but also clinically ineffective. His skepticism stemmed from the lack of antiseptic techniques and the high risk of infection and postoperative complications [12].

Despite rejecting tracheostomy, Hippocrates provided alternative methods for managing airway obstruction. He advocated for positional therapy, in which patients were reclined or adjusted in a way that facilitated airflow. He also prescribed steam inhalation, dietary modifications, and herbal remedies to alleviate respiratory distress. While these approaches did not provide immediate relief in cases of acute obstruction, they reflected the philosophy of gradual and systemic healing that characterized Hippocratic medicine [17].

Although Hippocratic texts contain no direct evidence that tracheostomy was performed or recommended, later Greek physicians, particularly during the Hellenistic and Roman periods, challenged Hippocratic opposition and began to explore airway interventions more extensively.

### 6.2. The Legendary Tracheotomy of Alexander the Great

One of the most fascinating, albeit anecdotal, references to airway intervention in ancient Greece is linked to Alexander the Great (356–323 BCE). According to historical accounts, particularly those attributed to Homerus of Byzantium, Alexander is said to have performed a life-saving incision on the battlefield, using the tip of his sword to open the trachea of a suffocating soldier [16,17,20,56,64].

While the authenticity of this account remains uncertain, the story highlights the rudimentary understanding of airway management that may have existed in military and survival contexts. The observation that neck wounds sometimes resulted in temporary airway patency might have influenced later physicians to consider surgical interventions for airway obstruction. Although this was far from a controlled surgical procedure, the legend of Alexander’s battlefield tracheotomy suggests that practical knowledge of airway intervention existed outside formal Greek medical practice [17,65].

This account also parallels later battlefield medicine practices, where emergency tracheostomies were often performed using improvised techniques. The idea that military necessity could drive surgical innovation is a theme that recurs throughout medical history, from ancient Greece to modern wartime medicine.

### 6.3. Asclepiades of Bithynia and Early Tracheostomy Attempts

During the Hellenistic period, one of the first Greek physicians to seriously consider tracheostomy as a viable procedure was Asclepiades of Bithynia (c. 124–40 BCE). Practicing in Rome, Asclepiades rejected the strictly conservative Hippocratic approach and introduced a more interventionist philosophy based on the principles of painless medicine and gentle therapies [24]. For many scholars the first recorded tracheostomy is attributed to Asclepiades of Bithynia. He was reportedly the first to perform a transverse incision through the trachea to facilitate air passage to the lungs. However, while this intervention was intended as a life-saving measure for airway obstruction, Asclepiades’ role in the development of tracheostomy is debated. Some scholars argue that he was not performing a true tracheostomy but rather engaging in procedures designed to clear phlegm and mucous secretions rather than mechanically opening the windpipe [24,66].

Unlike Hippocrates, Asclepiades believed that tracheotomy could be used as a non-emergency intervention to relieve airway obstruction. His approach reflected the broader shift in Hellenistic medicine, which embraced more experimental surgical procedures and greater anatomical knowledge. However, despite his pioneering ideas, tracheostomy was not widely adopted in his time, largely because the risks of infection and postoperative complications remained high [25]. Departing from the traditional Hippocratic humoral theory, Asclepiades proposed that diseases resulted from disruptions in the body’s atomic structure, specifically the movement and arrangement of corpuscles. His therapeutic approaches emphasized non-invasive treatments such as diet modifications, exercise, massage, and the use of wine. Asclepiades’ innovative medical practices, including his work in tracheotomy, significantly influenced subsequent Roman medical thought and laid foundational principles for future medical advancements [25,66].

Asclepiades’ contributions to airway surgery paved the way for later Roman and Byzantine physicians, who further explored the potential applications of tracheotomy in clinical practice.

### 6.4. Aretaeus of Cappadocia and the Risks of Tracheostomy

A highly influential physician of the second century CE, Aretaeus of Cappadocia documented various diseases of the respiratory system, including asthma, pneumonia, and bronchial obstructions. However, despite recognizing the theoretical benefits of tracheostomy, he ultimately discouraged its use due to the high risk of infection and poor wound healing [9,14,57,67,68].

Aretaeus wrote: ‘The lips of the wound do not coalesce, for they are both cartilaginous and not of a nature to unite’. This observation underscores a major anatomical challenge faced by early tracheostomy practitioners—the inherent difficulty of healing tracheal cartilage incisions without modern surgical suturing techniques [9].

Aretaeus’ caution mirrored Hippocratic concerns but, unlike Hippocrates, he documented tracheostomy as a theoretical possibility, suggesting that, by the second century CE, the procedure had at least been considered and debated in medical circles [9].

### 6.5. Antyllus and Surgical Refinements

One of the most significant advancements in tracheostomy techniques came from Antyllus, a Greek surgeon active in Rome during the second century CE. Unlike his predecessors, Antyllus actively performed and refined tracheostomy procedures, making his techniques closer to modern surgical standards. Although Antyllus’ original writings have not survived, his technique for tracheotomy—preserved through later sources such as Oribasius—appears to have been based on observations and refinements drawn from both anatomical study and likely clinical experience on human patients. Unlike Galen, Antyllus is generally believed to have performed tracheotomies on humans, as his descriptions include precise indications and postoperative care considerations consistent with human cases [26,68].

He recommended a transverse incision between the third and fourth tracheal rings, a method that remains similar to modern tracheostomy practices [26]. Antyllus also emphasized the importance of postoperative care to prevent complications such as infection. He identified cases where tracheostomy was ineffective, particularly in severe laryngotracheobronchitis, where the pathology extended beyond the operative site [57,68,69,70,71,72]. Ancient sources, particularly through the writings of Oribasius referencing Antyllus, describe the placement of tubular devices—sometimes referred to as ‘buttons’—into the tracheal opening, likely in human patients, to maintain airway patency. However, the exact timing and frequency of these applications remain uncertain, as detailed clinical records from antiquity are scarce.

### 6.6. Galen’s Contributions to Airway Anatomy and Artificial Ventilation

Among the most influential figures in ancient respiratory medicine, Galen of Pergamon (129–199 CE) played a pivotal role in advancing the understanding of airway anatomy and respiratory physiology. His extensive anatomical studies, primarily based on animal dissections—since human dissection was largely prohibited in his time—provided foundational insights that shaped medical knowledge for centuries. Although Galen made substantial contributions to the understanding of airway anatomy and respiratory physiology, his experiments—including artificial ventilation using bellows—were probably conducted exclusively on animals. There is no evidence to suggest that he ever performed a tracheotomy on a human. One of Galen’s most significant contributions was his demonstration that the larynx is responsible for voice production, an observation that refined the understanding of phonation and laid the groundwork for later studies in speech and respiratory function. By severing the recurrent laryngeal nerves of animals, he observed immediate vocal cord paralysis, definitively linking the larynx to voice modulation. This discovery not only advanced ancient knowledge of airway physiology but also had lasting implications for future research into laryngeal function and surgical interventions [28,32,73,74,75,76,77].

Beyond his contributions to airway anatomy, Galen conducted pioneering experiments in artificial ventilation, laying the foundation for the principles of mechanical respiration. He used bellows to inflate the lungs of a deceased animal, demonstrating that external forces could facilitate lung expansion even in the absence of spontaneous breathing. This experiment was groundbreaking, as it provided early empirical evidence that respiration was not solely an innate biological process but could be mechanically assisted—a concept that foreshadowed modern ventilatory support techniques. His findings suggested that the lungs functioned as passive organs that responded to external air pressure, a theory that influenced respiratory physiology well into the Renaissance [9,14,57,68].

In addition to his experimental work, Galen’s detailed descriptions of tracheal and bronchial anatomy refined the classical understanding of pulmonary structures. He identified the cartilaginous rings of the trachea, noting their role in maintaining airway patency and preventing collapse during respiration. His observations contributed to the later surgical approaches to airway obstruction, as physicians began to recognize the importance of maintaining an open airway in cases of respiratory distress. Moreover, his writings on the interplay between the diaphragm, intercostal muscles, and lung expansion introduced an early physiological framework for understanding the mechanics of breathing [19,75,77].

Galen’s work extended beyond the Greek medical tradition and profoundly influenced Arab, Byzantine, and Renaissance medicine. His texts, translated and studied extensively in the Islamic Golden Age, became foundational references for medieval physicians, including Avicenna (Ibn Sina) and Albucasis (Al-Zahrawi), who further developed airway management techniques based on his principles. Later, in the Renaissance period, Andreas Vesalius (1514–1564) challenged and refined Galenic anatomical descriptions, but Galen’s experimental approach to ventilation remained influential in shaping early respiratory interventions [78,79,80,81].

While Galen himself did not advocate for tracheotomy, his meticulous anatomical observations and artificial ventilation experiments indirectly contributed to the evolution of airway management. His pioneering research provided the intellectual framework upon which later physicians built, ultimately paving the way for modern tracheostomy procedures, mechanical ventilation, and respiratory medicine.

### 6.7. The Legacy of Byzantine Scholars in Preserving and Advancing Airway Surgery

Although Antyllus’ original writings did not survive, his surgical contributions were meticulously preserved and transmitted by later medical scholars, including Oribasius (c. 320–400 CE) and Paul of Aegina (c. 625–690 CE). Their efforts ensured that his knowledge influenced Byzantine, medieval, and even early Islamic surgical practices, forming a crucial link between ancient Greek medicine and later developments in the field [17,57,73,74].

Oribasius of Pergamon was a Byzantine Greek physician and personal physician to Emperor Julian the Apostate. He is best known for compiling the extensive *Synagoge Medica* (Medical Compendium), a vast 70-volume encyclopedia that preserved, systematized, and transmitted earlier Greek medical knowledge, including the works of Hippocrates, Galen, and Antyllus. Although Oribasius did not introduce new tracheotomy techniques, his detailed anatomical descriptions and compilations of prior surgical knowledge played a crucial role in preserving Galenic teachings on the airway and respiration. His encyclopedic work served as a foundational medical reference for Byzantine, medieval Islamic, and Renaissance scholars, helping to ensure the survival and continued evolution of surgical techniques, including airway management and tracheotomy procedures [73].

**Figure 3:**
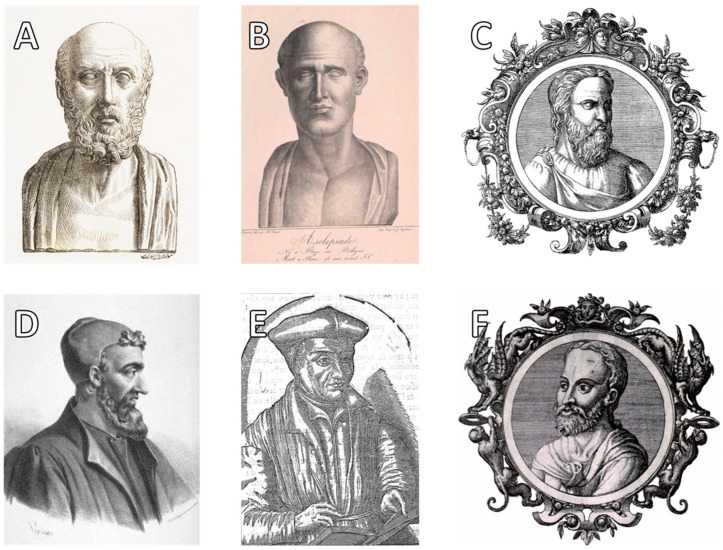
Key ancient, Hellenistic, and Byzantine Greek figures in the development and evolution of tracheotomy techniques. (**A**) Hippocrates of Kos (c. 460–370 BCE), (**B**) Asclepiades of Bithynia (c. 124–40 BCE), (**C**) Aretaeus of Cappadocia (c. 80–130 CE), (**D**) Galen of Pergamon (129–199 CE), (**E**) Oribasius of Pergamon (c. 320–400 CE), and (**F**) Paul of Aegina (c. 625–690 CE).

Paul of Aegina (Paulus Aegineta), another Byzantine Greek physician, is particularly renowned for his seven-volume medical encyclopedia, the ‘Epitome of Medicine’. This seminal work, written in the seventh century CE, compiled and expanded upon the accumulated surgical knowledge of his predecessors, including Antyllus, Galen, and Oribasius. Paul’s sixth book, which focused on surgery, became one of the most influential surgical treatises of the Middle Ages, widely studied by both Byzantine and Islamic physicians. In this work, he provided a detailed description of tracheotomy, discussing indications, anatomical considerations, and surgical techniques. His writings were particularly valued in the Islamic Golden Age, where scholars such as Al-Razi (Rhazes) and Al-Zahrawi (Albucasis) translated and further developed his surgical methods. Paul of Aegina’s legacy extended into the Renaissance, where his meticulous accounts of surgical procedures, including airway interventions, continued to influence the development of modern surgical techniques [74,75,76]. Figure 3 highlights the most influential Greek physicians who made significant contributions to the advancement of tracheotomy techniques across different historical periods.

## 7. Comparison with Modern Techniques

**Figure 4:**
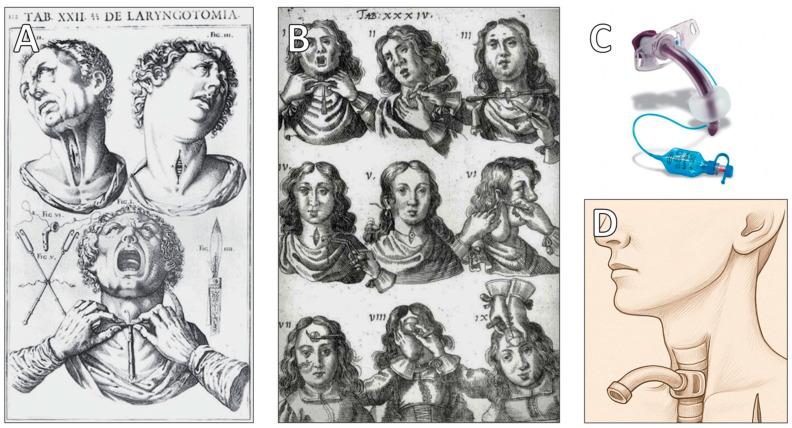
Evolution of tracheostomy from early anatomical descriptions to modern surgical practice. (**A**) Historical depiction of a ‘laryngotomia’ from *De Vocis Auditusque Organis Historia Anatomica* by Giulio Casseri (Julius Casserius, 1552–1616), one of the earliest documented visual representations of a tracheostomy procedure. (**B**) Engraving from *Armamentarium Chirurgicum Bipartitum* (1666), illustrating sequential steps of tracheotomy as practiced in the 17th century. This period, spanning from 1500 to 1833, recorded only 28 successful tracheotomies, as reported by Szmuk et al. (2008) [71]. Image courtesy of the U.S. National Library of Medicine. (**C**) Photograph of a modern tracheal cannula, showcasing the evolution of airway management devices and the technological advances that have transformed the safety and efficacy of tracheostomy procedures. (**D**) Schematic illustration of a modern tracheostomy, depicting a horizontal skin incision above the sternal notch and placement of a tracheostomy tube. This representation highlights the procedural precision and standardization achieved in contemporary surgical practice.

In ancient times, tracheostomy was performed under vastly different conditions than those of modern surgical practice. Given the high risk of complications and the rudimentary medical knowledge available, it is likely that tracheostomy was reserved for severe airway obstruction, where no other intervention could provide relief. The choice of incision location was also an important consideration. In situations where a long-term survival outcome was not expected, ancient physicians may have opted to make an incision between tracheal rings rather than directly into them, as this approach could have reduced the risk of excessive cartilage damage. The historical evolution of tracheostomy techniques—from early anatomical illustrations to modern procedural standards—is depicted in Figure 4, offering a visual comparison that highlights both the ingenuity of early practitioners and the advancements of contemporary surgical practice.

Classical authors were well aware of surgical interventions involving the esophagus, particularly in procedures designed to correct diverticula or stenoses, which required precise division of tissue. These techniques suggest that ancient physicians may have considered extending their knowledge to the trachea, though there is no direct evidence of tracheostomy being systematically performed. Despite this uncertainty, what is known is that central tracheal division became the method of choice for severe airway stenoses leading to life-threatening obstruction—a technique that remained unchanged until the twentieth century [24,82,83].

The materials and instruments available for tracheostomy in antiquity were far more primitive than those used today. Many surviving tracheostomy tubes from the classical period feature small circular forcing buttons at the tip rather than flaring flanges, which are now the standard design for modern tracheostomy tubes. These early designs suggest that there was no standardized method of coupling the tracheostomy tube to the patient’s airway until the introduction of studded brass tracheostomy tubes and gauze dressings in later centuries. Had modern decannulation technology been available in ancient times, tracheostomy would have been a far safer and more widely practiced procedure. Conversely, the absence of a specialized decannulation device may have limited the procedure’s use, particularly in cases where permanent tracheostomy was not required [24,83].

### 7.1. Evolution of Tracheostomy and Thoracic Surgery

In the surgical practices of antiquity, many procedures focused on the retention of substances within natural cavities rather than their mechanical evacuation. Emergency surgical procedures were often seen as last-resort measures, with thoracic drainage emerging as an alternative to relieve respiratory distress caused by secretion accumulation in the trachea. While aspiration and thoracic drainage were known techniques, the use of mechanical ventilation or suction in airway management remained largely unexplored [3,6,17,60].

Although Hippocrates remains the most celebrated figure in ancient medicine, the true origins of tracheostomy—as a procedure involving both incision into the trachea (tomy) and the placement of a device to maintain a functional stoma (stomy)—remain uncertain, due to limited documentation regarding when and how early physicians ensured prolonged airway patency. While later medical scholars attributed the procedure to Asclepiades, it is likely that rudimentary tracheal interventions predated his work. The concept of opening the airway to remove obstructions was known by the third century BCE, as evidenced by discussions on instruments designed to extract foreign bodies from the respiratory tract. However, whether these instruments were used specifically for tracheostomy or for more general airway clearance remains a matter of historical speculation [14,16,54,71].

### 7.2. Advancements in Technology and Procedure

Despite the historical significance of tracheostomy, there is limited information on the indications, timing, and technique of the procedure in antiquity. Most references to airway interventions describe obstruction due to mucus accumulation rather than surgical correction of stenosis or structural abnormalities. The limited application of tracheostomy in ancient times can be attributed to several factors. First, the shorter life expectancy and higher mortality rates from trauma, infections, and respiratory diseases meant that long-term management of airway stenosis was not a common medical concern. Additionally, acute laryngotracheitis was often attributed to atmospheric changes rather than intrinsic airway pathology, limiting the incentive for direct surgical intervention [6,16,54,72].

As medical knowledge evolved, anatomical, physiological, and pathological studies of the tracheobronchial system led to significant advancements in Greek medicine. The theory of Pneumatism, which emerged during this period as part of Gnosticism, introduced the idea that airflow through the trachea played a fundamental role in bodily function. This theory, which replaced earlier solidification-based models of physiology, suggested that air-contaminated disease mechanisms were responsible for respiratory conditions [61,84,85,86].

Prominent Greek physicians contributed to this understanding, with Eryximachos and Diokles making significant observations regarding tracheobronchial function and airflow mechanics. Eryximachos was among the first to theorize that airway function was linked to speech production and temperature regulation of inhaled gases. Diokles, another influential physician of the ancient Greek medical school, further explored pathological conditions affecting the tracheobronchial tree. He proposed that impaired air entry due to partial occlusion of the trachea could lead to respiratory insufficiency, and he suggested that pressure exerted on the pharynx and trachea could influence air passage [61,87,88,89,90].

These concepts were further refined by later Greek scholars, including Aretaeus of Cappadocia and Galen of Pergamon, who investigated the role of the trachea in respiratory diseases. Aretaeus’ writings on asthma, apnea, and airway pathology laid the foundation for future respiratory medicine, while Galen’s anatomical studies of the larynx and trachea significantly advanced airway surgery and mechanical ventilation concepts. Galen’s observation that the trachea functioned not only as a conduit for air but also as a site for voice modulation played a crucial role in shaping future airway interventions [19,61,67,77,91].

The evolution of tracheostomy and thoracic surgery in ancient Greece was a complex process influenced by anatomical discoveries, medical philosophies, and technological limitations. While the procedure itself was rarely performed, its theoretical foundations were established through progressive experimentation and evolving medical knowledge. The transition from Hippocratic opposition to the tentative acceptance of airway interventions in the Hellenistic and Roman periods highlights the gradual advancement of airway management techniques [3,9,17,72,89].

The absence of specialized surgical tools and decannulation devices hindered the widespread adoption of tracheostomy in ancient times, but Greek physicians made remarkable contributions to understanding respiratory anatomy and pathology. The work of Eryximachos, Diokles, Aretaeus, and Galen laid the groundwork for modern respiratory and airway management techniques, demonstrating how ancient medical theories continue to influence contemporary surgical practice [6,54,61].

## 8. Legacy and Modern Implications of Ancient Greek Tracheostomy

Tracheostomy was first practiced in ancient Greece, long before it became a common surgical procedure and a milestone in the evolution of thoracic surgery and medicine. The findings of this study highlight the timeless significance of medical innovations, particularly in the management of respiratory diseases and airway obstruction. Understanding the historical development of operative procedures is crucial for appreciating the clinical and surgical methods that are used today. The evolution of tracheostomy reflects the gradual progression of medical knowledge, demonstrating how ancient surgical innovations have influenced contemporary medical practice. Recognizing the historical context of life-saving surgical techniques allows for a deeper appreciation of modern advancements, emphasizing the continuous refinement of medical and surgical technology. This procedure, which stands as one of the earliest recorded surgical interventions, has played a pivotal role in the history of respiratory medicine, offering life-saving solutions for patients at risk of suffocation [27,51,92,93,94]. While ancient and early modern physicians—including Galen—experimented with artificial ventilation using methods such as bellows, the systematic use of tracheotomy as an access point for positive-pressure ventilation was not realized until 1952, during the polio epidemic in Copenhagen, when Bjørn Ibsen introduced prolonged manual ventilation via tracheostomy, laying the foundation for modern intensive care medicine, a development that stands in stark contrast to the experimental and primarily anatomical use of tracheotomy in antiquity [95].

### Relevance to Modern Medical Practice

The historical analysis of tracheostomy and its surgical management in classical Greece reveals an integrated medical approach that was based on early anatomical knowledge, surgical materials, and principles of asepsis. The classical Greek period (5th–4th century BCE) played a foundational role in shaping Western medicine, and its influence extends into contemporary medical practice. This study examines the Hippocratic Corpus, one of the most comprehensive medical texts from antiquity, identifying sections that contain critical observations on tracheostomy as well as discussions on the treatment of traumatic complications. Hippocrates emphasized that each case must be assessed individually, recognizing that factors such as infection risk, wound healing, and anatomical variations played a role in determining the success or failure of surgical interventions [2,7,22,72].

The surgical techniques and philosophical principles proposed by Hippocrates, particularly his systematic approach to anatomy and dissection of deceased individuals, were as revolutionary as the intellectual and philosophical advancements of his time. His scientific methodology reshaped the course of medicine, shifting it from a mystical and religious practice to an empirical, observation-based discipline. Hippocrates’ unwavering dedication to the pursuit of medical knowledge remains one of the most influential legacies in the history of science [7,68,90,94].

By analyzing his writings, medical ethics, and innovative surgical methods, this study underlines the lasting impact of Hippocratic medicine on modern surgical practice, particularly in tracheostomy management and airway surgery. His work continues to offer valuable lessons applicable to contemporary medicine, particularly in the areas of clinical decision-making, patient safety, and surgical ethics. The integration of ancient Greek medical wisdom with modern medical advancements highlights the universal and timeless nature of medical innovation, proving that the foundations of modern medicine were laid thousands of years ago [9,14,54,60,72].

## 9. Conclusions

The historical exploration of tracheostomy in ancient Greece reveals a fascinating intersection of medical ingenuity, anatomical discovery, and surgical evolution. While the procedure was not widely practiced due to Hippocratic opposition and technical limitations, its conceptual development laid the groundwork for later advancements in thoracic surgery and airway management. The contributions of Greek physicians such as Asclepiades, Aretaeus, Antyllus, and Galen highlight a gradual shift in medical thought, from skepticism toward surgical intervention to a more systematic exploration of airway procedures. The archaeological and literary evidence examined in this study demonstrates that ancient Greek medicine was not only theoretical but also deeply practical, striving to balance empirical knowledge with ethical considerations. As modern medicine continues to evolve, understanding these early advancements provides valuable historical perspective on the progression of surgical techniques, reinforcing the timeless pursuit of medical innovation and patient care.

## Data Availability

The data presented in this study are available upon request from the corresponding author.
